# Clinical Characteristics of Dizzy Patients Showing Discordant Results Between Bithermal Caloric Test and Video Head Impulse Test

**DOI:** 10.3390/jcm14124350

**Published:** 2025-06-18

**Authors:** Hahn Jin Jung, Sangeun Lee, Hyeop Oh, Jee Hye Wee, Chang Gun Cho, Joo Hyun Park

**Affiliations:** 1 Department of Otorhinolaryngology-Head and Neck Surgery, Chungbuk National University College of Medicine, Chungbuk National University Hospital, Cheongju 28644, Republic of Korea; hahnjin2@naver.com; 2 Department of Otorhinolaryngology-Head and Neck Surgery, College of Medicine, Dongguk University, Ilsan Hospital, Goyang 10326, Republic of Korea; selee1006@dgu.ac.kr (S.L.); oholoto@dgu.ac.kr (H.O.); cho69@dumc.or.kr (C.G.C.); 3 Department of Otorhinolaryngology-Head and Neck Surgery, Hallym University Sacred Heart Hospital, Hallym University College of Medicine, Anyang 14068, Republic of Korea; weejh07@hanmail.net; 4 Sensory Organ Research Institute, College of Medicine, Dongguk University, Gyengju 38066, Republic of Korea

**Keywords:** caloric test, dizziness, dissociation, peripheral vestibular disorder, vestibular function test, video head impulse test

## Abstract

**Background/Objectives:** To evaluate the clinical characteristics and diagnostic significance of dissociation between bithermal caloric test and video head impulse test (vHIT) in patients presenting with dizziness. **Methods**: We retrospectively reviewed 644 patients who underwent bithermal caloric testing and vHIT at a university-affiliated general hospital. Patients were classified into concordant and discordant groups based on test results. The discordant group was further subdivided into those with abnormal caloric test and normal vHIT, and those with normal caloric test and abnormal vHIT. Demographic data, vestibular function test outcomes, and clinical diagnoses were analyzed. **Results**: Discordant results were observed in 36.5% of patients. Among these, 31.8% had abnormal caloric responses with normal vHIT, and 4.7% had normal caloric responses with abnormal vHIT. Most patients in both discordant subgroups were diagnosed with peripheral vestibular disorders, such as Ménière’s disease and unilateral vestibulopathy. The abnormal caloric/normal vHIT pattern was more common and associated with low-frequency dysfunction. The normal caloric/abnormal vHIT pattern, though less frequent, also involved predominantly peripheral etiologies. **Conclusions**: Dissociation between caloric and vHIT results is not uncommon and provides important diagnostic insights. Employing both tests in a complementary manner enhances the identification of frequency-specific vestibular deficits and supports more accurate diagnosis and management of vestibular disorders.

## 1. Introduction

The bithermal caloric test and video head impulse test (vHIT) are two essential vestibular function tests commonly used in the assessment of dizzy patients [1]. The caloric test is traditionally regarded as the gold standard for evaluating unilateral vestibular weakness due to its sensitivity in detecting low-frequency vestibulo-ocular reflex (VOR) abnormalities via thermal convection stimulation [2]. Specifically, it assesses the function of the horizontal semicircular canals, which are innervated by the superior vestibular nerve. On the other hand, vHIT evaluates high-frequency VOR responses by measuring eye movements during rapid, unpredictable head impulses, thus providing a more physiological assessment of semicircular canal function during daily activities [3]. Caloric testing and vHIT each provide distinct but complementary insights into vestibular function. Caloric testing evaluates extremely low-frequency vestibular responses and is particularly useful for detecting subtle interaural asymmetries in vestibular function, which may not be captured by high-frequency tests such as vHIT [4]. In contrast, vHIT assesses high-frequency responses, more closely mimicking everyday head movements. It is less affected by external and middle ear conditions and enables assessment of all semicircular canals, including vertical canals (anterior and posterior), which indirectly allows evaluation of both superior and inferior vestibular nerve function. Generally, these tests are expected to yield concordant results, where both tests are either normal or abnormal in a patient. However, clinical experience and recent studies have increasingly reported discordant results, wherein one test appears abnormal while the other remains normal, a phenomenon referred to as dissociation [5,6,7].

This dissociation between caloric test and vHIT outcomes has drawn considerable attention, as it carries significant diagnostic and pathophysiological implications. Previous studies have reported that discordance occurs in a substantial portion of vestibular patients, ranging from approximately 20% to 40%, emphasizing the necessity for clinicians to interpret these discrepancies accurately [6,7]. Previous research has suggested various underlying mechanisms for these dissociations, notably frequency-dependent vestibular dysfunction, differential vulnerability of type I and type II vestibular hair cells, and structural changes such as endolymphatic hydrops [8,9,10].

Clinically, dissociated patterns have been reported with differing etiological associations [8,9,11]. Patients presenting with an abnormal caloric test and normal vHIT frequently have peripheral lesions, notably Ménière’s disease and chronic vestibular neuritis. Conversely, those with abnormal vHIT and normal caloric test results have traditionally been associated more closely with central lesions. However, recent studies demonstrate that peripheral pathologies such as acute vestibular neuritis and Ménière’s disease may also present abnormal vHIT with normal caloric test outcomes, challenging the conventional perspectives and necessitating reconsideration of diagnostic approaches [12,13].

Recent larger-scale studies have begun to provide deeper insights into the phenomenon of dissociation between vestibular tests. A study involving 2101 patients demonstrated significant dissociation between caloric and vHIT test outcomes, reporting that approximately 25% of patients exhibited dissociated results, highlighting the diagnostic utility of these patterns in differentiating conditions such as Ménière’s disease from vestibular migraine [14]. Another large-scale analysis of 893 dizzy patients revealed a dissociation rate of approximately 18.1%, predominantly seen in peripheral vestibular disorders such as Ménière’s disease and vestibular neuritis [15]. These recent findings have provided deeper insights into the frequency, clinical relevance, and disease-specific patterns of dissociation.

In this retrospective study, we aimed to further evaluate the clinical characteristics of patients exhibiting discordant results between caloric test and vHIT. By analyzing a substantial patient cohort from a university-affiliated general hospital, we sought to elucidate the frequency of discordance, associated demographic factors, underlying etiologies, and diagnostic implications of such dissociation. The findings from this study could significantly refine clinical assessment protocols, contribute to a deeper understanding of vestibular pathophysiology, and provide clinicians with practical guidelines for interpreting discordant vestibular function test outcomes.

## 2. Materials and Methods

### 2.1. Study Design and Population

This study was conducted as a retrospective review of medical records of patients who underwent bithermal caloric test and vHIT from May 2019 to December 2022 at a Dongguk University, Ilsan hospital. Collected data included patient demographics (age, sex), clinical history, and detailed results from vestibular function tests including bithermal caloric test (canal paresis values, maximum slow-phase velocity) and vHIT (horizontal canal (HC) VOR gain, presence of overt/covert saccades, and abnormal findings in anterior canal (AC)/posterior canal (PC) when applicable). In selected patients, additional audiovestibular assessments such as pure-tone audiometry, cervical/ocular vestibular-evoked myogenic potentials (cVEMP/oVEMP), and brain/internal auditory canal MRI were reviewed. Final clinical diagnoses were determined based on standard diagnostic criteria. The inclusion criteria were patients who had dizziness and underwent both caloric tests and vHIT within a 1-week interval. Exclusion criteria included patients with incomplete test results, other central nervous system disorders confirmed by imaging, previous otologic surgery, or a history of vestibulotoxic drug use. In addition, all patients underwent routine otoscopic examination before vestibular testing. Individuals with tympanic membrane perforation or other conditions precluding safe caloric irrigation were excluded from caloric testing and thus not included in the study. This study was approved by the Institutional Review Board of Dongguk University Ilsan Hospital (IRB No. 2025-05-004).

### 2.2. Vestibular Function Tests

Caloric test was performed using a water caloric irrigator (ICS Chartr 200, GN Otometrics, Denmark) with temperatures set at 30 °C for cold irrigation and 44 °C for warm irrigation. Each irrigation lasted 30 s, with a 5 min interval between irrigations. The induced nystagmus responses were recorded and analyzed using video-oculography. Maximum slow phase velocity of caloric nystagmus was measured on each irrigation. Unilateral vestibular weakness was defined as caloric paresis of 25% or greater, calculated using Jongkees’ formula [16].

The vHIT was performed using an ICS Impulse device (GN Otometrics, Taastrup, Denmark). All vHIT tests were performed by a single experienced examiner to minimize interobserver variability. Patients wore goggles equipped with a small video-oculography camera to record eye movements. Patients were seated 1 m from a fixation point, and the examiner passively and randomly rotated the patient’s head approximately 20 times to the left or right side with a low amplitude (10–20°) and high peak velocity (150–250°/s). Repeat measurements were performed as needed to ensure consistency and waveform reliability. Only the gain of HC vHIT results was considered in this study, with gains below 0.8 considered abnormal. In this study, only HC gain values were used to define abnormal vHIT results. Cases showing saccade-like waveforms with normal gain were excluded from analysis. Additionally, patients with normal HC vHIT gain but with overt and/or covert saccades, as well as patients with abnormal caloric test and normal HC vHIT plus abnormal AC or PC results, were additionally reviewed.

### 2.3. Group Classification

Based on the results of caloric test and vHIT, patients were divided into two primary groups. The concordant group included those whose results were either both normal or both abnormal. The discordant group included patients showing mismatched results between the two tests and was further classified into two subgroups: those with abnormal caloric test results (paresis ≥ 25%) and normal vHIT gain (≥0.8), and those with abnormal vHIT gain (<0.8) and normal caloric test results (paresis < 25%).

### 2.4. Clinical Diagnoses

Vestibular neuritis (VN) was diagnosed as a first-ever spontaneous vertigo lasting longer than 24 h with spontaneous horizontal and torsional nystagmus to the abnormal side, without hearing loss or central neurological signs [17]. VN patients were also classified by the timing of the test into acute (<5 days after symptom onset), subacute (5–14 days), and chronic (>14 days) phases. Unilateral vestibulopathy was defined as unilateral loss of VOR function persisting for 3 or more months.

Other vestibular disorders were diagnosed based on standard clinical guidelines: Benign paroxysmal positional vertigo (BPPV) according to the AAO-HNSF guideline (2017) [18]; Ménière’s disease (MD) by the Bárány Society (2015) [19]; vestibular migraine by the Bárány Society (2012) [20]; bilateral vestibulopathy by Bárány Society (2017) [21]; presbyvestibulopathy by the Bárány Society (2019) [22]; benign paroxysmal vertigo by ICHD-3 (2018) [23]; and persistent postural-perceptual dizziness (PPPD) by Staab et al. (2017) [24]. Sudden sensorineural hearing loss (SSNHL) with vertigo, cerebellopontine angle (CPA) tumors, other central diseases, and undetermined cases were also included. CPA tumor cases were analyzed as part of the central disease category.

### 2.5. Statistical Analysis

Statistical analyses were conducted using SPSS software (version 25.0; SPSS Inc., Chicago, IL, USA). Descriptive statistics were used to summarize patient demographics and clinical data. Normality of data was tested using the Shapiro–Wilk test. Chi-square tests were applied to evaluate associations between categorical variables, while continuous variables were analyzed using the independent *t*-test or Mann–Whitney U test as appropriate. A *p*-value less than 0.05 was considered statistically significant.

## 3. Results

A total of 644 patients met the inclusion criteria and were included in the final analysis. Among them, 409 patients (63.5%) exhibited concordant results between caloric test and vHIT, with 305 patients (47.4%) showing normal results in both tests and 104 patients (16.1%) showing abnormalities in both tests. Discordant results were observed in 235 patients (36.5%), including 205 patients (31.8%) with abnormal caloric test and normal vHIT, and 30 patients (4.7%) with normal caloric test and abnormal vHIT.

**Figure 1:**
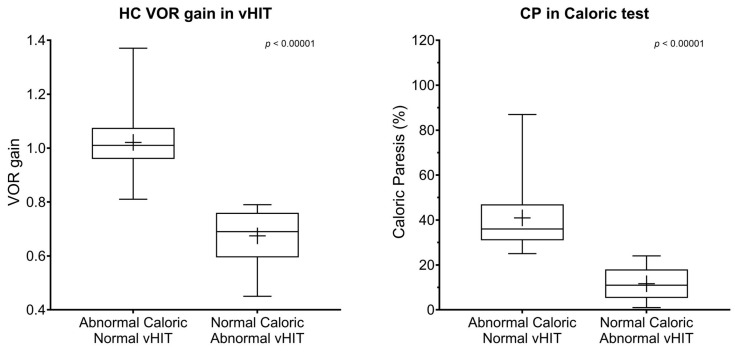
Horizontal canal VOR gain and caloric paresis between the two discordant groups. Boxes indicate the interquartile range, with the central horizontal line representing the median. Whiskers extend to the minimum and maximum values. The plus sign (+) indicates the mean of each group. HC, horizontal canal; VOR, vestibule-ocular reflex; CP, canal paresis. *p* < 0.05 was considered statistically significant.

Patients with abnormal caloric test and normal vHIT (n = 205) had a mean age of 52.87 ± 17.42 years, while those with normal caloric test and abnormal vHIT (n = 30) had a significantly higher mean age of 65.70 ± 13.26 years (*p* < 0.001). The sex distribution was not statistically different between groups (M:F = 91:114 in the abnormal caloric group vs. 12:18 in the abnormal vHIT group; *p* = 0.153). The affected side distribution (right:left:bilateral) was 109:69:27 in the abnormal caloric group and 14:16:0 in the abnormal vHIT group (*p* = 0.021) (Table 1, Figure 1).

In the group with abnormal caloric test and normal vHIT, the mean VOR gain in HC vHIT was 1.02 ± 0.11 and the mean caloric paresis was 40.23 ± 17.42%. In contrast, the group with normal caloric test and abnormal vHIT had a significantly lower mean VOR gain of 0.75 ± 0.12 and a lower CP value of 11.67 ± 7.8% (both *p* < 0.001) (Table 1).

Among the 205 patients in the abnormal caloric/normal vHIT group, 189 patients (92.2%) were diagnosed with peripheral vestibular disorders, including unilateral vestibulopathy (n = 90), involving HC hypofunction on vHIT, Ménière’s disease (n = 40), bilateral vestibulopathy (n = 21), vestibular neuritis/labyrinthitis (n = 18), BPPV (n = 8), SSNHL with vertigo (n = 5), vestibular migraine (n = 2), presbyvestibulopathy (n = 1), and others (n = 3). Only 9 patients (4.4%) had central lesions and 1 patient was undetermined. In the normal caloric/abnormal vHIT group (n = 30), 25 patients (83.3%) were diagnosed with peripheral vestibular disorders, including unilateral vestibulopathy (n = 10), BPPV (n = 4), MD (n = 3), VN/labyrinthitis (n = 2), SSNHL with vertigo (n = 3), vestibular migraine (n = 1), Presby vestibulopathy (n = 1), and undetermined (n = 1). Central lesions were identified in 5 patients (16.7%) (Table 2).

## 4. Discussion

In this study, we investigated the clinical characteristics and diagnostic implications of dissociation between caloric test and vHIT in dizzy patients. Among the 644 patients included, discordant results were observed in over one-third of cases, with abnormal caloric response and normal vHIT being the more prevalent pattern (31.8% vs. 4.7%).

The dissociation most frequently observed in our study—abnormal caloric test with normal vHIT—is consistent with previously published literature, which attributes this pattern to the fundamental difference in stimulus frequency between the two tests [8,9,10]. The dissociation patterns observed may reflect underlying frequency-dependent dysfunction, differential involvement of type I versus type II vestibular hair cells, or central compensation mechanisms [7,14]. However, further mechanistic validation through neuroimaging or vestibular evoked potentials would be needed in future studies to clarify these associations. Caloric test stimulates the VOR at ultra-low frequencies, while vHIT evaluates high-frequency responses. As a result, patients with selective impairment of low-frequency VOR pathways, such as those with endolymphatic hydrops or chronic vestibular neuritis, may exhibit abnormal caloric responses despite preserved vHIT gain. The predominance of unilateral vestibulopathy and Ménière’s disease in this group supports this interpretation.

Conversely, patients with normal caloric test results and abnormal vHIT findings represented a smaller portion of our cohort. This dissociation pattern has been considered likely of central origin in prior studies, although mixed etiologies are possible [5,11,15]. For example, studies have reported that lesions in the cerebellum—particularly involving the flocculus or nodulus—can impair high-frequency VOR responses while sparing low-frequency pathways, resulting in abnormal vHIT with preserved caloric function [8]. Furthermore, infarcts in the medial vestibular nucleus or brainstem pathways have also been associated with such dissociation [11]. Some authors have proposed that central compensation mechanisms following central vestibular insult may selectively recover low-frequency VOR, mimicking this test discrepancy [15]. In this study, patients with normal caloric/abnormal vHIT results were significantly older than those with abnormal caloric/normal vHIT results. However, the majority of patients in this group were diagnosed with peripheral vestibular disorders. This discrepancy may be due in part to our patient population, which was primarily drawn from an otolaryngology clinic. It is also possible that excluding corrective saccades from the abnormality criteria led to underrecognition of certain vHIT abnormalities, potentially affecting the observed dissociation rate.

Overall, our findings emphasize the importance of utilizing both caloric tests and vHIT as complementary tools in the clinical evaluation of dizziness. While caloric testing offers high sensitivity to low-frequency vestibular dysfunction and helps quantify canal paresis, vHIT provides critical insight into high-frequency dynamic VOR responses during physiologic head movement. Together, they allow for a more comprehensive assessment of semicircular canal function across broad frequency ranges [25]. This study focused solely on HC vHIT gain to ensure a valid comparison with caloric test data. Therefore, AC and PC function was not included in the main analysis, as the study’s aim was not to provide a comprehensive assessment of all semicircular canals, but to highlight the potential discrepancy in test outcomes when evaluating the same canal with two distinct physiological stimuli. We acknowledge that excluding vertical canal findings may have overlooked cases of isolated AC or PC dysfunction. For instance, PC hypofunction is commonly observed in SSNHL with vertigo, likely due to shared vascular supply between the cochlea and PC. Similarly, selective vertical canal deficits are characteristic of inferior vestibular neuritis or certain central lesions. Future prospective studies incorporating full three-canal vHIT analysis could provide deeper pathophysiological insights, particularly in these subgroups.

In a supplementary correlation analysis, we evaluated the relationship between vHIT gain asymmetry and CP. After excluding bilateral cases, we observed a moderate correlation across the entire cohort (r = 0.585, *p* < 0.0001). This association was particularly strong in the concordant group—comprising patients with either “both normal” or “both abnormal” results (r = 0.749, *p* < 0.0001). As expected, the discordant group—which included patients with “abnormal caloric/normal vHIT” and “normal caloric/abnormal vHIT” results—did not exhibit a significant correlation (r = 0.024, *p* = 0.741). These findings suggest that gain asymmetry and CP reflect overlapping but distinct physiological dimensions of vestibular function, especially when test results diverge. For instance, in acute VN, caloric tests may remain normal while vHIT reveals hypofunction early in the disease course. Furthermore, because both caloric testing and HC vHIT primarily reflect superior vestibular nerve function, this study was not able to identify cases of inferior vestibular neuritis, which may involve isolated PC dysfunction. Conversely, in conditions like Ménière’s disease, caloric responses are often attenuated due to hydropic effects on thermal convection, despite preserved high-frequency responses on vHIT. Our study supports this complementary utility by showing that 31.8% of patients had abnormal caloric test results with normal vHIT, many of whom were diagnosed with predominantly peripheral disorders such as Ménière’s disease and unilateral vestibulopathy. This aligns with previous reports demonstrating preserved high-frequency function in certain peripheral lesions [25]. Meanwhile, although dissociation involving abnormal vHIT and normal caloric responses is classically associated with central lesions, the majority of our patients with this pattern also had peripheral diagnoses, indicating that test interpretation must be contextualized based on the clinical setting. It should be noted that the current study defined abnormal vHIT solely based on HC gain values below 0.8, without including isolated saccadic responses. While this approach enhanced consistency across the dataset, it may have led to underestimation of compensated or subtle vestibular dysfunction. Future prospective investigations with real-time evaluation of overt and covert saccades may help refine the interpretation of dissociation between caloric test and vHIT.

Relying solely on one test may overlook frequency-specific dysfunction or compensation effects, leading to incomplete or even misleading diagnostic impressions. Combined interpretation of both tests enables more accurate localization, pathophysiologic insight, and phase-specific understanding of vestibular pathology, ultimately enhancing diagnostic precision and informing appropriate treatment strategies. Previous studies have demonstrated that the correlation between caloric testing and vHIT varies depending on the underlying vestibular disorder. For instance, in Ménière’s disease, caloric responses are often attenuated even when vHIT remains normal, suggesting that caloric testing may be more sensitive in early stages [4,26]. When evaluating patients with VM and MD, approximately 12% of patients showed an abnormal vHIT and a normal caloric response, while about 34% exhibited an abnormal caloric test and a normal vHIT [26]. In contrast, vestibular neuritis typically presents with abnormalities in both tests. In patients with vestibular schwannoma, significant inverse correlation between CP and VOR gain has been observed [27]. These findings underscore the need for disease-specific interpretation when evaluating discordant or concordant test results. The recognition of dissociation patterns may also assist in clinical decision-making. When discordant findings are identified, clinicians should consider detailed history-taking to assess for prior episodes of vertigo, auditory symptoms, and fluctuating disease course. For instance, abnormal caloric responses with normal vHIT gain can appear in early-stage Ménière’s disease, but may be indistinguishable from compensated vestibular neuritis or central causes. Follow-up evaluation using audiometry, electrocochleography, or inner ear MRI imaging may help elucidate the underlying diagnosis. Longitudinal monitoring for fluctuating or progressive symptoms is essential to guide clinical management.

This study has several limitations. First, its retrospective design may introduce selection bias. Furthermore, the exclusion of patients with prior otologic surgery, central nervous system lesions, or vestibulotoxic drug history may have led to underrepresentation of certain co-existing vestibular disorders. In addition, comparative subgroup analysis between concordant and discordant groups was not performed in the main analysis. Compared to the overall cohort, the abnormal caloric/normal vHIT group demonstrated a higher proportion of peripheral vestibular disorders, whereas the normal caloric/abnormal vHIT group showed a relatively greater proportion of central disorders. Nevertheless, given that this study was conducted in an otolaryngology-based patient population, peripheral vestibular disorders were the predominant diagnoses across all groups. In addition, since a detailed diagnostic review was not systematically performed for all patients in the concordant group due to incomplete follow-up documentation and variability in diagnostic coding, subgroup comparisons should be interpreted with caution.

Second, not all patients underwent adjunctive testing such as VEMPs or MRI, potentially affecting diagnostic precision. Similarly, SVV testing was not consistently performed, and standardized follow-up intervals were not ensured, limiting assessment of long-term compensation. Third, analysis was restricted to HC vHIT data. Inclusion of vertical canal information could have enhanced diagnostic value. Additionally, although several patients met caloric criteria for bilateral vestibular loss, none exhibited bilateral vHIT hypofunction by gain criteria in our dataset. This may reflect the limitation of using HC gain alone, without incorporating saccade analysis or vertical canal data. Moreover, several patients met the caloric criteria for bilateral vestibular hypofunction but exhibited preserved HC gain on vHIT. Although these cases technically satisfied the Bárány Society definition, the lack of consistent follow-up and the retrospective nature of limited documentation of chronic vestibular symptoms, make it difficult to confirm true bilateral vestibulopathy. As a result, the prevalence of bilateral vestibular loss in this study may have been overestimated, and findings should be interpreted with caution. Furthermore, corrective saccades were not included in the vHIT abnormality criteria, possibly leading to underestimation of subtle deficits. Directional preponderance (DP) values were not analyzed, although they may provide useful diagnostic clues in suspected central lesions. Future studies should consider integrating DP with CP analysis for more comprehensive interpretation. Lastly, the unequal sample sizes between discordant subgroups may limit the statistical power of group comparisons and should be considered when interpreting the results.

## 5. Conclusions

In this study, we investigated the clinical characteristics and diagnostic implications of dissociation between caloric test and vHIT in dizzy patients. These findings underscore the need for incorporating both caloric tests and vHIT in a complementary fashion to achieve more accurate and comprehensive vestibular diagnoses. By leveraging the strengths of each test, clinicians can better identify frequency-specific deficits and improve diagnostic confidence across a broad spectrum of vestibular disorders.

## Figures and Tables

**Table 1:** Demographics and vestibular test parameters.

	Abnormal Caloric/Normal vHIT (n = 205)	Normal Caloric/Abnormal vHIT (n = 30)	*p*-Value
Age (years)	52.87 ± 17.42	65.70 ± 13.26	<0.001
Sex (M:F)	91:114	12:18	0.153
Side (R:L:B)	109:69:27	14:16:0	0.021
VOR gain	1.02 ± 0.11	0.75 ± 0.12	<0.001
CP (%)	40.23 ± 17.42	11.67 ± 7.8	<0.001

VOR, vestibulo-ocular reflex; CP, canal paresis. *p* < 0.05 was considered statistically significant.

**Table 2:** Etiologic distribution of discordant groups.

	Abnormal Caloric/Normal vHIT (n = 205)	Normal Caloric/Abnormal vHIT (n = 30)
Unilateral vestibulopathy	90	10
Ménière’s disease	40	3
Bilateral vestibulopathy	21	0
Vestibular neuritis/labyrinthitis	18	2
Acute phase	7	0
Subacute phase	9	2
Chronic phase	2	0
BPPV	8	4
SSNHL with vertigo	5	3
Vestibular migraine	2	1
Presbyvestibulopathy	1	1
Other peripheral disorders	3	0
Other central lesion	7	4
Undetermined	1	1

BPPV, benign paroxysmal positional vertigo; SSNHL, sudden sensorineural hearing loss.

## Data Availability

The data presented in this study are available upon request from the corresponding author due to the privacy of patient information.

## References

[1] Bery A.K., Hale D.E., Newman-Toker D.E., Saber Tehrani A.S. (2025). Evaluation of Acute Dizziness and Vertigo. Med. Clin. N. Am..

[2] van de Berg R., Rosengren S., Kingma H. (2018). Laboratory examinations for the vestibular system. Curr. Opin. Neurol..

[3] Rosengren S.M., Young A.S., Taylor R.L., Welgampola M.S. (2022). Vestibular function testing in the 21st century: Video head impulse test, vestibular evoked myogenic potential, video nystagmography; which tests will provide answers?. Curr. Opin. Neurol..

[4] Molnár A., Maihoub S., Tamás L., Szirmai Á. (2023). Comparison between caloric and video-head impulse tests in Ménière’s disease and vestibular neuritis. Int. J. Audiol..

[5] Sinha S.K., Neupane A.K., Gururaj K. (2025). Dissociation between caloric test and the video head impulse test in individuals with auditory neuropathy spectrum disorders. J. Laryngol. Otol..

[6] Tamanini J.B., Mezzalira R., Vallim M.G.B., Gabriel G.P., Stoler G., Chone C.T. (2023). Dissociation between video head impulse test and caloric test: A marker of Ménière’s disease?—A systematic review and meta-analysis. Braz. J. Otorhinolaryngol..

[7] Waissbluth S., Sepúlveda V. (2022). Dissociation between Caloric and Video Head Impulse Tests in Dizziness Clinics. Audiol. Res..

[8] Kim H.S., Oh E.H., Kim J.Y., Choi S.Y., Choi K.D., Choi J.H. (2022). Discordant vestibulo-ocular reflex function according to the frequency and mode of stimulation. J. Neurol..

[9] McCaslin D.L., Rivas A., Jacobson G.P., Bennett M.L. (2015). The dissociation of video head impulse test (vHIT) and bithermal caloric test results provide topological localization of vestibular system impairment in patients with “definite” Ménière’s disease. Am. J. Audiol..

[10] Cerchiai N., Navari E., Dallan I., Sellari-Franceschini S., Casani A.P. (2016). Assessment of Vestibulo-oculomotor Reflex in Ménière’s Disease: Defining an Instrumental Profile. Otol. Neurotol..

[11] Li X., Ling X., Li Z., Song N., Ba X., Yang B., Yang X., Sui R. (2025). Clinical characteristics of patients with dizziness/vertigo showing a dissociation between caloric and video head impulse test results. Ear. Nose Throat J..

[12] Redondo-Martínez J., Bécares-Martínez C., Orts-Alborch M., García-Callejo F.J., Pérez-Carbonell T., Marco-Algarra J. (2016). Relationship between video head impulse test (vHIT) and caloric test in patients with vestibular neuritis. Acta Otorrinolaringol. Esp..

[13] Zellhuber S., Mahringer A., Rambold H.A. (2014). Relation of video-head-impulse test and caloric irrigation: A study on the recovery in unilateral vestibular neuritis. Eur. Arch. Otorhinolaryngol..

[14] Mavrodiev V., Strupp M., Vinck A.S., van de Berg R., Lehner L. (2024). The dissociation between pathological caloric testing and a normal video head impulse test helps differentiate between Ménière’s disease, vestibular migraine, and other vestibular disorders: A confirmatory study in a large cohort of 2,101 patients. Front. Neurol..

[15] Lee J.Y., Kwon E., Kim H.J., Choi J.Y., Oh H.J., Koo J.W., Kim J.S. (2020). Dissociated Results between Caloric and Video Head Impulse Tests in Dizziness: Prevalence, Pattern, Lesion Location, and Etiology. J. Clin. Neurol..

[16] Jongkees L.B.W. (1948). Value of the caloric test of the labyrinth. Arch. Otolaryngol..

[17] Strupp M., Bisdorff A., Furman J., Brandt T., Kim J.S., Straumann D., Jahn K., Newman-Toker D.E., Lempert T. (2022). Acute unilateral vestibulopathy/vestibular neuritis: Diagnostic criteria. J. Vestib. Res..

[18] von Brevern M., Bertholon P., Brandt T., Fife T., Imai T., Nuti D., Newman-Toker D.E. (2017). Benign paroxysmal positional vertigo: Diagnostic criteria Consensus document of the Committee for the Classification of Vestibular Disorders of the Bárány Society. Acta Otorrinolaringol. Esp..

[19] Lopez-Escamez J.A., Carey J., Chung W.H., Goebel J.A., Magnusson M., Mandalà M., Newman-Toker D.E., Strupp M., Suzuki M., Trabalzini F. (2015). Diagnostic criteria for Ménière’s disease. J. Vestib. Res..

[20] Lempert T., Olesen J., Furman J., Waterston J., Seemungal B., Carey J., Bisdorff A., Versino M., Evers S., Newman-Toker D. (2012). Vestibular migraine: Diagnostic criteria. J. Vestib. Res..

[21] Strupp M., Kim J.S., Murofushi T., Straumann D., Jen J.C., Rosengren S.M., Della Santina C.C., Kingma H. (2017). Bilateral vestibulopathy: Diagnostic criteria Consensus document of the Classification Committee of the Bárány Society. J. Vestib. Res..

[22] Agrawal Y., Van de Berg R., Wuyts F., Walther L., Magnusson M., Oh E., Sharpe M., Strupp M. (2019). Presbyvestibulopathy: Diagnostic Criteria. Consensus Document of the Classification Committee of the Bárány Society. J. Vestib. Res..

[23] (2018). The International Classification of Headache Disorders 3rd Edition. https://ichd-3.org/.

[24] Staab J.P., Eckhardt-Henn A., Horii A., Jacob R., Strupp M., Brandt T., Bronstein A. (2017). Diagnostic criteria for persistent postural-perceptual dizziness (PPPD): Consensus document of the committee for the Classification of Vestibular Disorders of the Bárány Society. J. Vestib. Res..

[25] Melville I.Z., Yamsuan K., Wu H., Thorne P.R., Kobayashi K., Taylor R.L. (2024). Do measures of gain asymmetry and catch-up saccades improve video head impulse test agreement with caloric results?. Clin. Neurophysiol. Pract..

[26] Yilmaz M.S., Egilmez O.K., Kara A., Guven M., Demir D., Genc Elden S. (2021). Comparison of the results of caloric and video head impulse tests in patients with Meniere’s disease and vestibular migraine. Eur. Arch. Otorhinolaryngol..

[27] Brown C.S., Peskoe S.B., Risoli T., Garrison D.B., Kaylie D.M. (2019). Associations of Video Head Impulse Test and Caloric Testing among Patients with Vestibular Schwannoma. Otolaryngol. Head Neck Surg..

